# Development of Gold-Nanoparticle-Based Lateral Flow Immunoassays for Rapid Detection of TB ESAT-6 and CFP-10

**DOI:** 10.3390/bios13030354

**Published:** 2023-03-06

**Authors:** Palesa Pamela Seele, Busiswa Dyan, Amanda Skepu, Charlotte Maserumule, Nicole Remaliah Samantha Sibuyi

**Affiliations:** 1 Nanotechnology Innovation Centre, Health Platform, Advanced Materials Division, Mintek, Private Bag X3015, Randburg, Johannesburg 2125, South Africa; 2 Advanced Chemistry and Life Sciences Division, Next Generation Health Cluster, Council for Scientific and Industrial Research (CSIR), Pretoria 0001, South Africa

**Keywords:** tuberculosis, rapid diagnostic test, point of care, immunoassay, ESAT-6, CFP-10

## Abstract

The current study reports on the development of a rapid and cost-effective TB-antigen diagnostic test for the detection of *Mycobacterium* biomarkers from non-sputum-based samples. Two gold nanoparticle (AuNP)-based rapid diagnostic tests (RDTs) in the form of lateral flow immunoassays (LFIAs) were developed for detection of immunodominant TB antigens, the 6 kDa early secreted antigen target EsxA (ESAT-6) and the 10 kDa culture filtrate protein EsxB (CFP-10). AuNPs were synthesized using the Turkevich method and characterized by UV-vis spectrophotometer and transmission electron microscope (TEM). The AuNP–detection probe conjugation conditions were determined by comparing the stability of 14 nm AuNPs at different pH conditions, following salt challenge. Thereafter, ESAT-6 and CFP-10 antibodies were conjugated to the AuNPs and used for the colorimetric detection of TB antigens. Selection of the best detection and capture antibody pairs was determined by Dot spotting. The limits of detection (LODs) for the LFIAs were evaluated by dry testing. TEM results showed that the 14 nm AuNPs were mostly spherical and well dispersed. The ESAT-6 LFIA prototype had an LOD of 0.0625 ng/mL versus the CFP-10 with an LOD of 7.69 ng/mL. Compared to other studies in the literature, the LOD was either similar or lower, outperforming them. Moreover, in some of the previous studies, an enrichment/extraction step was required to improve on the LOD. In this study, the LFIAs produced results within 15 min and could be suitable for use at PoCs either in clinics, mobile clinics, hospitals or at home by the end user. However, further studies need to be conducted to validate their use in clinical samples.

## 1. Introduction

Tuberculosis (TB) is an infectious disease caused by *Mycobacterium tuberculosis* (*M*. *tb*), which when detected timeously and with appropriate therapeutic intervention can be curable. The global representation of this crisis is massive, as 3.6 million of TB cases can go undiagnosed or unreported in low–middle income countries. In sub-Saharan Africa, 5 out of 10 active TB cases go undiagnosed, and in South Africa (SA) alone, an estimated 150,000 of cases are not diagnosed, which poses a risk for increasing the rates of transmission, morbidity and mortality [1]. The lack of rapid, accurate diagnostic tools at a point of care (PoC), including the inaccessibility of easily collectible samples, is central to the missed diagnosis and subsequent spread of the disease. In the current study, the immunodominant TB antigens ESAT-6 and CFP-10 were explored as targets for the development of TB-antigen rapid diagnostic test (RDT) prototypes which were based on the lateral flow immunoassays (LFIAs) technology.

To date, microbiological testing such as sputum smear microscopy and sputum culture test remain the most widely used methods of TB diagnosis [2]. The major limitation of the sputum smear microscopy test which exploits staining of acid-fast bacteria (AFB) is that it requires 10,000 bacilli/mL from a sputum sample for a positive TB detection [2], making it less sensitive for paucibacillary cases in HIV positive patients, children who often present with extra-pulmonary TB (EPTB) and very ill patients who cannot expectorate [2,3,4]. The sputum culture test has a long turnaround time of 2 to 4 weeks. These tests are expensive and need to be carried out by experienced personnel in specialized facilities and with costly instrumentation. Likewise, molecular tests such as the PCR-based GeneXpert MTB/RIF (Cepheid Inc., Sunnyvale, CA, USA) and the TB loop-mediated isothermal amplification (LAMP) (Eiken; Tokyo, Japan) are costly, and not readily accessible to low-income countries nor suitable for PoC testing [5,6,7]. The blood-based immune response tuberculin skin test (TST) and the IFN-γ release assays (IGRAs) (QuantiFERON-TB Gold, Cellestis Ltd., Australia) have uncircumventable limitations such as their inability to accurately distinguish between active and latent infections [4]. In addition, TST does not differentiate *M*. *tb* from nontuberculous *Mycobacterium* and the Bacille Calmette–Guérin (BCG) vaccine, making it unsuitable for TB testing in a South African cohort [2]. 

Lateral flow immunoassays are user friendly, produce results within 10 to 15 min and are suitable for PoC use either at medical facilities or at home by the end user [8,9]. The Alere Determine™ LFIA was introduced in South Africa (SA) in 2018, and used to detect the *Mycobacterial* lipoarabinomannan (LAM) antigen in urine samples within 25 min. This test, however, was found to be more suitable for TB testing in HIV-positive patients. The commercially available LFIA-based TB RDTs have limited endorsement from the World Health Organization (WHO) [10], and in some studies, the reported sensitivity and specificity of the diagnostic kits were inconsistent [11]. The poor specificity may be attributed to cross-reactivity with the BCG vaccine and other *Mycobacterium* species [12]. 

**Scheme 1:**
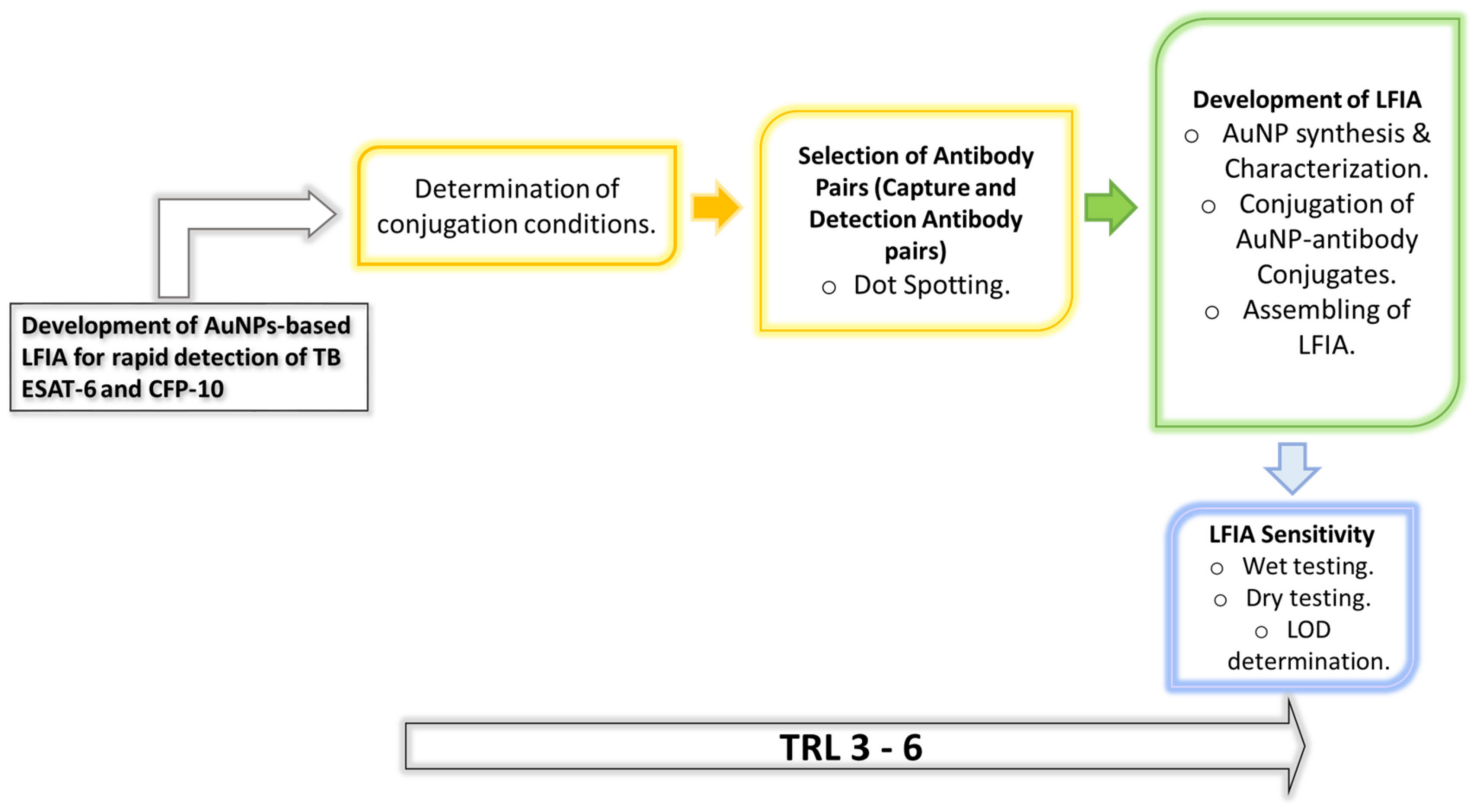
Simplified representation of the research strategy. The diagram depicts the objectives of the research work.

In contrast, the current TB-antigen RDT explores the immunodominant TB secreted antigens, ESAT-6 and CFP-10, as targets in detecting TB following the research strategy summarized in Scheme 1. In the globally used BCG vaccine, these antigens are not expressed subsequent to the deletion of the region of difference (RD)1 locus in which they are located [13,14]. As a result, cross-reactivity in patients previously vaccinated with BCG and/or those infected with other *Mycobacterium species* will be minimized [15,16,17]. Additionally, these biomarkers have been previously detected in the serum from HIV-positive and HIV-negative pulmonary TB (PTB) and extra-pulmonary TB (EPTB) patients with similar sensitivities. The antigens were detectable at low concentrations <10 nM in the serum, which was useful for diagnosis and evaluating anti-TB response [14]. To expedite the Stop TB Strategy, TB RDTs are now adopted to detect TB biomarkers in easily accessible samples such as urine [8] and blood samples, and sensing of volatile organic compounds from the breath [9,10]. The presence of ESAT-6 and CFP-10 in urine [15,16,17,18] and blood [14,15] samples makes them ideal TB biomarkers, especially for RDTs. However, the methods used to detect antigens such as mass spectrometry (MS) and ELISA are not suitable for POC testing, as the very long sample processing and analysis require highly trained personnel. Therefore, the proposed AuNP-based LFIAs will be optimized for rapid detection of ESAT-6 and CFP-10 from non-sputum samples which will be beneficial for patients who cannot expectorate.

## 2. Materials and Methods

### 2.1. Materials

The ESAT-6 and CFP-10 antibodies were obtained from manufacturers outlined in Table 1. The antibodies were given specific codes in the current study, as indicated in the table. The gold (III) chloride (HAuCl_4_.3H_2_O), trisodium citrate (Na_3_C_6_H_5_O_7_), and anti-Mouse IgM (μ-chain specific) antibody produced in goat were bought from Sigma-Aldrich (St Louis, Missouri, USA). The anti-Rabbit Affinity purified Goat IgG was acquired from R&D systems (Minneapolis, Minnesota, USA). The nitrocellulose membrane CN95 was purchased from Sartorius (PTY) LTD (Midrand, South Africa), whilst the Millipore conjugate pad G041 and Millipore C083 absorbent pad were bought from Sigma-Aldrich and the backing cards from Diagnostic Consulting Network (Carlsbad, California, USA). 

### 2.2. Synthesis of 14 nm AuNPs

The 14 nm AuNPs were synthesized using the Turkevich method (Turkevich et al., 1951), which entails the reduction of HAuCl_4_ with trisodium citrate as described previously. Trisodium citrate and HAuCl_4_.3H_2_O solutions were prepared at a concentration of 1% and 0.0289 M, respectively. The solutions were each filtered using 0.2 µm filters and thereafter used in the synthesis of the 14 nm AuNPs [15,16].

### 2.3. Characterization of the AuNPs Using UV-vis Spectrophotometer and TEM

The optical properties of the 14 nm AuNPs were measured using the UV-vis spectrophotometer (Multiskan^TM^ GO plate reader (Thermo Fischer Scientific, Waltham, Massachusetts, USA)) within a wavelength range of 200 to 800 nm. Transmission electron microscopy (TEM) analysis was carried out on the JEOL JEM-2100F following previous procedures [19,20].

### 2.4. Conjugation of Antibodies to AuNPs

#### 2.4.1. Stability of AuNP–Antibody Conjugates

Stability of 14 nm AuNP–antibody conjugates was assessed at different pH levels. The pH levels of AuNPs were adjusted to pH 7, 8, 9 and 10 using 0.1 M K_2_CO_3_. Thereafter, 100 µL of each AuNP solutions was dispensed into a 96-well plate. The antibodies were added to the AuNPs to a final concentration of 10 µg/mL. In negative controls, phosphate-buffered saline (PBS) was added in place of the antibodies. The reactions were incubated for 5 min at room temperature, then 50 µL of 0.1 M NaCl was added and color changes, if any, were recorded.

#### 2.4.2. Conjugation of Antibodies to the AuNPs

To conjugate the antibodies to the AuNPs, 1 mL of AuNP solutions (OD 1 for the 14 nm AuNPs) at the selected optimal pH and 1 µg/mL antibody were added to an Eppendorf tube and incubated for 20 min with agitation. Then, 100 µL of conjugate blocking buffer (10% bovine serum albumin (BSA) prepared in borate buffer, pH 9) was added to the conjugate mixture and further incubated for 30 min. The conjugate mixture was then centrifuged at 15 000 rpm for 20 min at 4 °C. The supernatant was discarded and the AuNPs were re-suspended in diluent buffer and stored at 4 °C until further analysis.

### 2.5. Selection of Antibody Pairs by Dot Spotting

The detection and capture antibody pairs for ESAT-6 and CFP-10 were tested for compatibility using dot spotting following a sandwich assay. Immobilization of antibodies was carried out by dot spotting 1 µL of 1 mg/mL of antibodies at about 6 mm of spacing between the control and the test line antibody. Thereafter, the membranes were dried for 15 min in an oven at 37 °C. Blocking of the membranes was carried out for 5 min in 0.1% BSA blocking buffer and dried for 45 min at 37 °C in an oven. Strips were cut into 3 mm and analyzed accordingly. Binding of the detection antibodies to the antigen was analyzed by immersing the strips in an Eppendorf tube containing the AuNP–antibody conjugate with buffer (negative test) or AuNP–antibody conjugate with buffer containing antigen (test sample) (refer to Scheme 1). The experiment was allowed to run for 15 min, and signals were then visualized by a red dot.

### 2.6. Development of the AuNP-Based LFIA and Determination of the Limit of Detection (LOD)

The selected control and test line antibodies were dispensed using XYZ (X = 25 mm; Y = 30 mm and Z = 8 mm) Platform Dispenser HM3030 (Shanghai Kinbio Tech.Co, LTD, Pudong New Strict, Shanghai, China) at a rate of 1 µL/cm on to the nitrocellulose membrane which was laminated onto a backing card. The card was then dried for 30 min at 37 °C in an oven, and the membrane was blocked with 0.1% BSA in phosphate buffer for 5 min with agitation. Drying was carried out for 45 min at 37 °C.

**Scheme 2:**
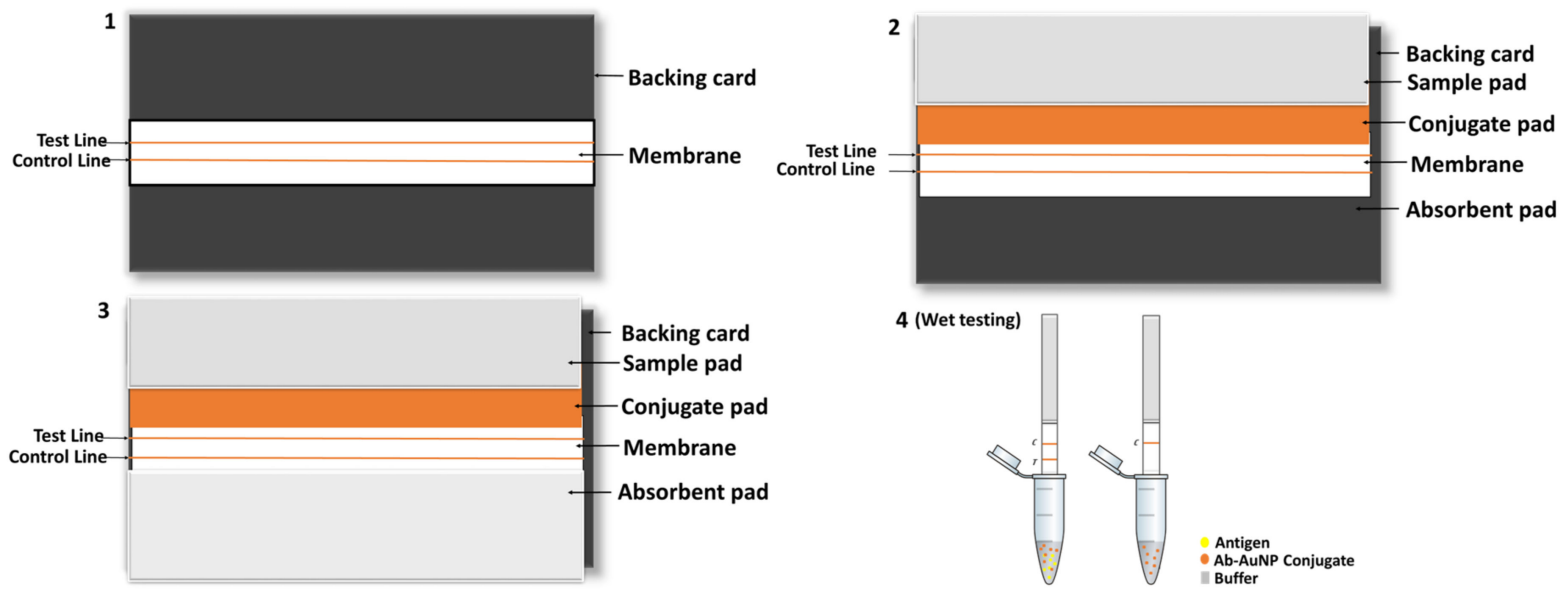
Schematic diagram showing steps of the assembling and wet testing of LFIA strips. The diagram illustrates the lamination of components of a strip on the backing card (steps 1 to 4). (**1**) The nitrocellulose membrane is the first to be laminated, followed by (**2**) the conjugate and sample pads which have a 2 mm overlap, and finally (**3**) the absorbent pad is embedded on the card. Following the cutting of the strips, wet testing (step 4) was carried out as shown on the diagram. Lines on both control and test lines represent a positive test, while a single line in the control represent a negative test.

Meanwhile, the conjugate pad was prepared by immersing it in conjugate pad blocking buffer (3% BSA in borate buffer) for 15 min with gentle agitation. Once the conjugate pad had been dried, the AuNP conjugate of choice was sprayed using the KinBio Dispenser instrument (Kinbio Tech.Co, LTD, Pudong New Strict, Shanghai, China) and again dried for 45 min at 37 °C. Finally, the conjugate pad and absorbent pad were laminated on the backing card with the membrane attached and cut into 3 mm strips using the High Speed Cutter ZQ4500 (Kinbio Tech.Co, LTD, Pudong New Strict, Shanghai, China) prior to assembling into cassettes (schematic representation of the assembling process in Scheme 2).

To determine the LOD of the prototypes, the recombinant antigens were serially diluted with 1 x PBS at concentrations ranging between 0.0769 to 0.0769 × 10^6^ ng/mL for CFP-10, and 0.0000625 to 62.5 × 10^2^ ng/mL for ESAT-6. The recombinant antigens were provided at different concentrations by suppliers; hence, the starting concentrations dictated the working solution’s dilution series. A volume (65 µL) of the prepared samples was loaded onto the assembled test strips and allowed to run for 15 min prior to the analysis of the results.

### 2.7. Wet and Dry Conjugate Testing of the LFIA

*Wet testing*: To perform wet conjugate testing, 30 µL of running buffer and 10 µL of AuNP–antibody conjugate were added to an Eppendorf tube. For a test sample, commercial recombinant *M*. *tb* CFP-10 (227-20144, Biodisc Raybiotech Life, Inc., Peachtree Corners, Georgia, USA) or *M*. *tb* ESAT-6 (Creative diagnostics PTY (Ltd), Shirley, NY, USA) was also added to separate tubes, after which the sample pad part of the strip was cut and immersed into those tubes. The test was allowed to run for 15 min prior to analysis.

*Dry testing*: The backing card was assembled with the conjugate pad which contained the AuNP–antibody conjugate. To conduct a negative test, 40 µL of running buffer (without any antigens/proteins) was added to the sample pad on the test strip and allowed to flow for 15 min. The same procedure was followed for a test sample by using a buffer containing the commercial recombinant proteins/antigens. Various concentrations of antigen were tested until a red line/dot was visible. Concentrations of 4 and 2.7 µg/mL for CFP-10 and ESAT-6, respectively, were used as a reference point, as determined in the Dot spotting results. Where necessary and where false negatives were observed higher concentrations were also tested.

## 3. Results

### 3.1. Synthesis and Characterization of AuNPs

**Figure 1:**
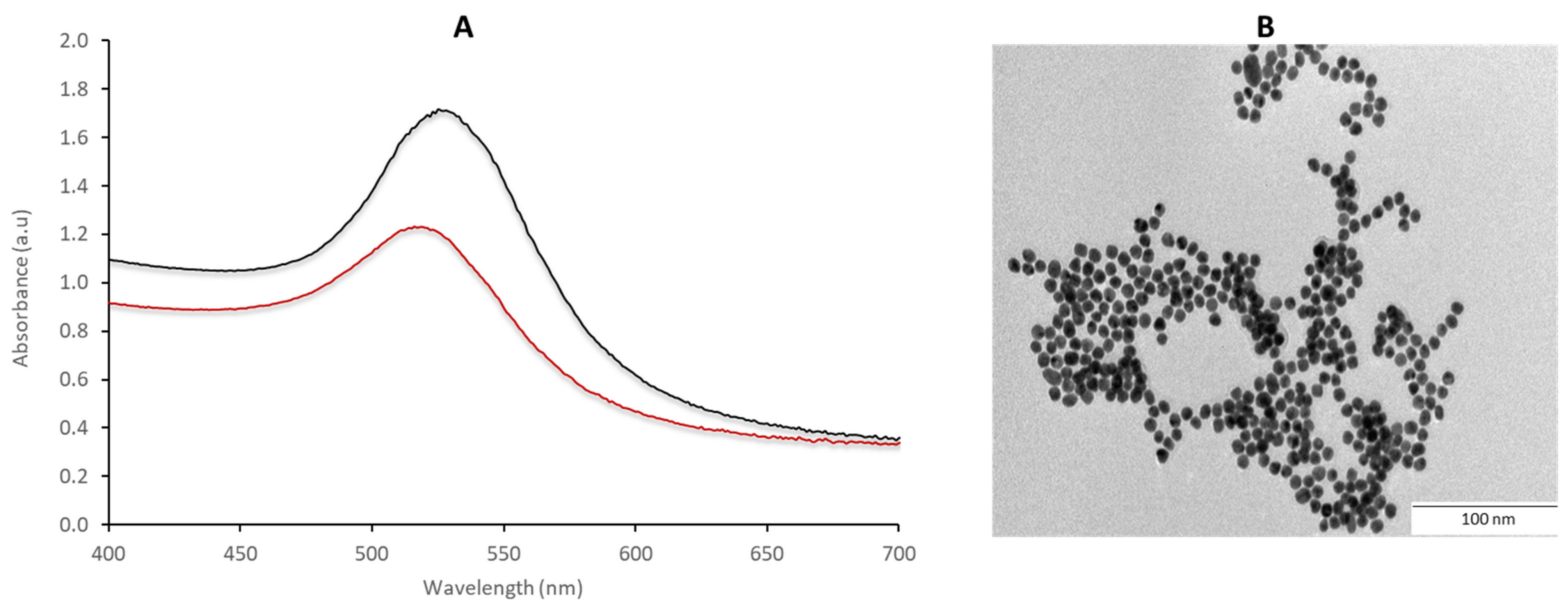
Characterization of 14 nm AuNPs. The nanoparticles were analyzed using (**A**) absorbance spectroscopy and (**B**) TEM. The absorption maxima were at 512 nm.

UV-vis spectroscopy and TEM were used to evaluate the quality of the synthesized AuNPs. The absorption maxima of the 14 nm AuNPs was at 519 nm (Figure 1A), which is a typical wavelength banding pattern for AuNPs owing to the surface plasmon resonance (SPR) [17,18,19,20]. The morphology of the AuNPs was mostly spherical, as shown by the TEM micrographs (Figure 1B), with an average core size of 14 nm. Using this method, different sized diameters of the AuNPs, in the range of 14 to 150 nm, can be produced by varying the ratio of the reducing agent to the gold precursor (trisodium citrate: gold chloride) [16,20,21]. AuNPs are able to absorb and scatter light, which enables them to emit different colors depending on their size, shape and degree of aggregation. This SPR phenomenon is known to occur due to the ability of the metal to conduct electrons simultaneously excited by electromagnetic waves striking the metal surface [19].

### 3.2. Assessment of Conjugation Conditions and Conjugate Stability

**Figure 2:**
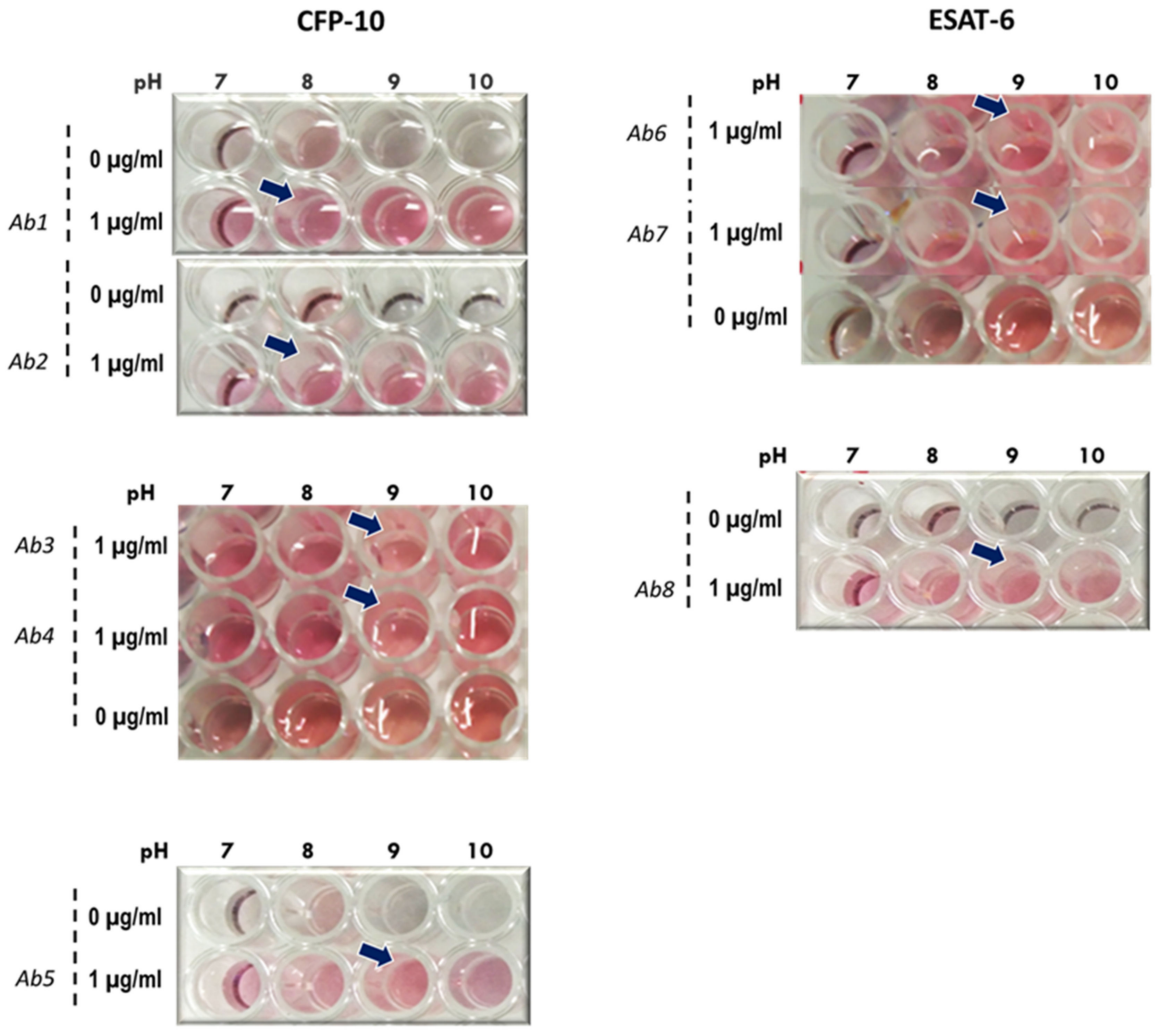
Stability of AuNPs with ESAT-6 and CFP-10 antibodies at different pH values. AuNPs of 14 nm at pH 7, 8, 9 and 10 were dispensed into wells of a 96-well plate. Anti-ESAT-6 and anti-CFP-10 antibodies were added into wells and incubated at room temperature for 5 min prior to addition of NaCl. Color changes, if any, were observed and with reference from AuNPs that had no antibody and NaCl, and optimum conditions for conjugation were chosen. The arrows indicate the selected conditions for conjugation.

The stability of the AuNP–antibody conjugates was visually assessed by color change following the addition of 0.1 M NaCl to the conjugate samples. AuNPs that had no salt nor antibody were used as a reference for unstable conjugates. In cases where color changes were not obvious by visual inspection, a pH that was closer to the protein’s storage buffer (prepared by the suppliers) was selected. The 14 nm AuNP–antibody conjugates that were indicative of instability changed color from red to purple or colorless, while the stable conjugates remained red after addition of NaCl. The color changes were as a result of flocculation after adding NaCl to AuNPs; under the tested conditions, this was more evident in the AuNP samples without antibodies (Figure 2). The selected pH for conjugation of Ab1 and Ab2 was pH 8, while Ab3, Ab4 and Ab5 were more stable at pH 9. Similarly, Ab6, Ab7 and Ab8 were stable at pH 9. In the current study, the conjugates were mostly stable at pH 8 and 9 with 14 nm AuNPs, and these were selected as the conjugation conditions for producing the detection probes (antibody–AuNP conjugates).

### 3.3. Selection of Antibodies

**Figure 3:**
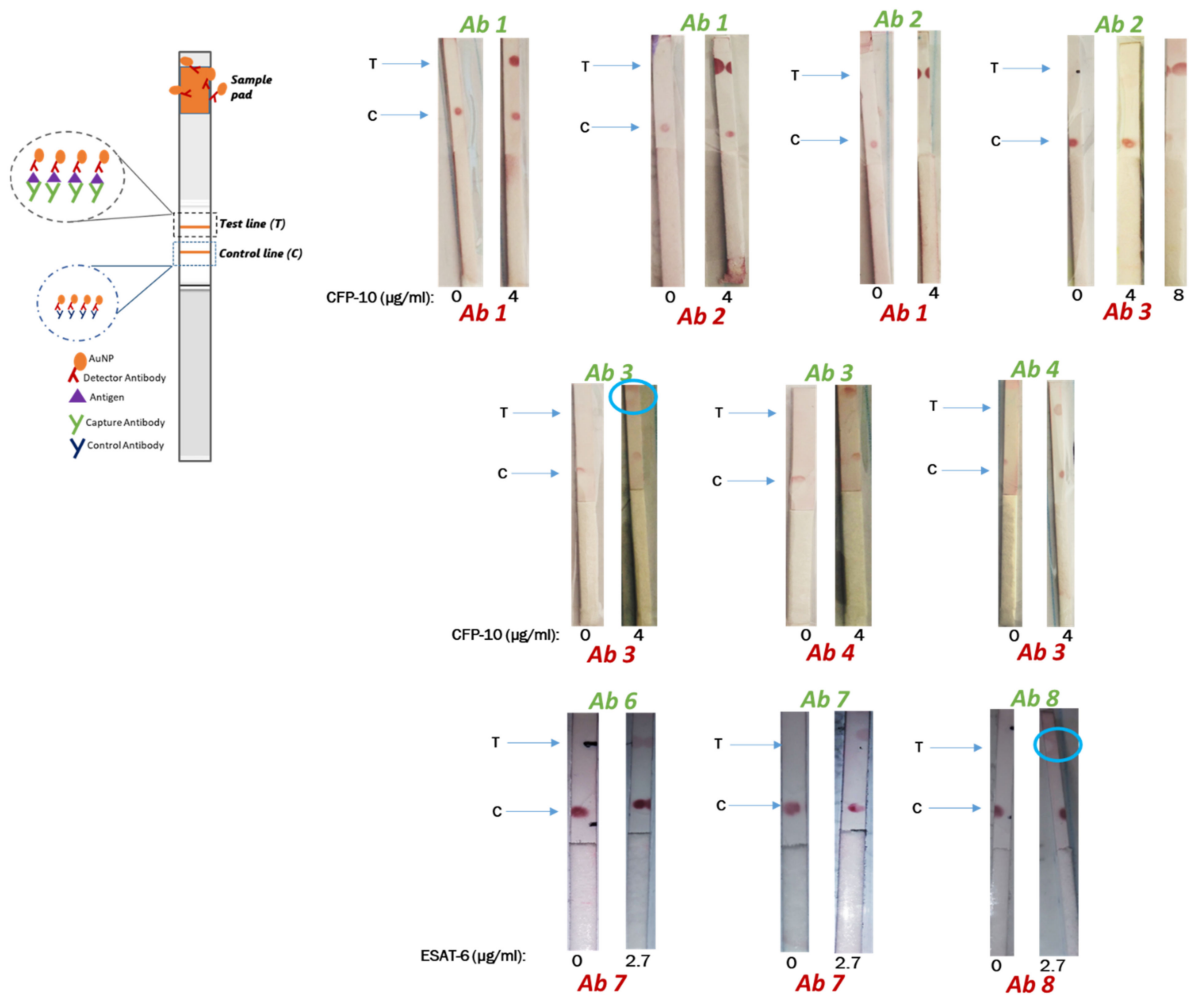
Compatibility of CFP-10 capture and detection antibody pairs. The capture and control antibodies were dotted on the nitrocellulose membrane using 1 µL of 1 mg/mL antibody. The membranes were left to dry for 15 min prior to testing. To test the samples, the negative control contained 30 µL of running buffer and 10 µL of AuNP–antibody conjugate, whilst the test sample also contained recombinant CFP-10 protein (4 µg/mL) or ESAT-6 protein (2.7 µg/mL). Tests were allowed to run for 15 min before analysis. ‘Ghost lines’ are indicated by blue circles.

Pairing and compatibility of the antibodies was evaluated by dot spotting to determine the capture and detection antibodies specific to CFP-10 and ESAT-6. Overall, CFP-10 had eight pairs of compatible capture and detection antibodies, whilst ESAT-6 had only two pairs to choose from (results are summarized in Table 2 and representative strips shown in Figure 3). With respect to CFP-10, the detection AuNP–antibody conjugates that were compatible with the Ab1 capture antibody were Ab1, Ab2 and Ab4, whilst the Ab2 capture antibody was compatible with the Ab1 and to a lesser extent with Ab3 and Ab4 AuNP–antibody conjugates as detection antibodies. Testing the pairs Ab2/Ab3 (capture antibody/detection antibody) and Ab2/Ab4 with double the concentration (from 4 to 8 µg/mL) of the recombinant CFP-10 antigen resulted in very faint dots at the control test and a concentrated dots at the test line.

Consistent with other studies, pairing the same antibody yielded unfavorable results, mostly false negatives, and also a ‘ghost line’ was depicted by the Ab3/Ab3 pair. ‘Ghost lines’ are an artefact that is characterized by a red background encircling a white dot where an antibody was immobilized. In contrast, the Ab1/Ab1 and Ab7/Ab7 pairs gave distinct red-colored dots with respect to the negative and test samples, and the Ab4/Ab4 showed a visible but faint dot upon testing with recombinant protein.

The Ab3 and Ab4 capture antibodies paired favorably with the Ab4 and Ab3 as detection antibodies, respectively. Ab5 (anti-CFP-10 antibody) and Ab8 (anti-ESAT-6 antibody) antibodies are rabbit polyclonal antibodies (pAb) that could not be paired with the mouse antibodies due to incompatibility with the secondary antibody at the control line. The Ab5/Ab5 pair gave false negatives whilst the Ab8/Ab8 gave false positives when testing BSA-based buffers. pAb targets multiple epitopes on an antigen, and this wide range makes them less specific when compared to monoclonal antibodies (mAb). Consequently, pAbs are less preferred as capture antibodies, and are rather used as detection antibodies.

The ESAT-6s Ab6 and Ab7 showed compatibility when paired in both orientations. Buffer components also influenced the antibody pairing outcomes. In most cases, BSA-based buffers gave false positives, such as when testing the Ab8/Ab8 pair between BSA concentrations of 0.1 to 1%. Although casein-based buffers were able to eliminate the false positivity, the Ab8 test line could not detect the recombinant protein. ‘Ghost lines’ were observed on the strips with the Ab3/Ab3 and with Ab8/Ab8 pairs where Tris-based buffers were used for optimization.

### 3.4. LOD for CFP-10 and ESAT-6

**Figure 4:**
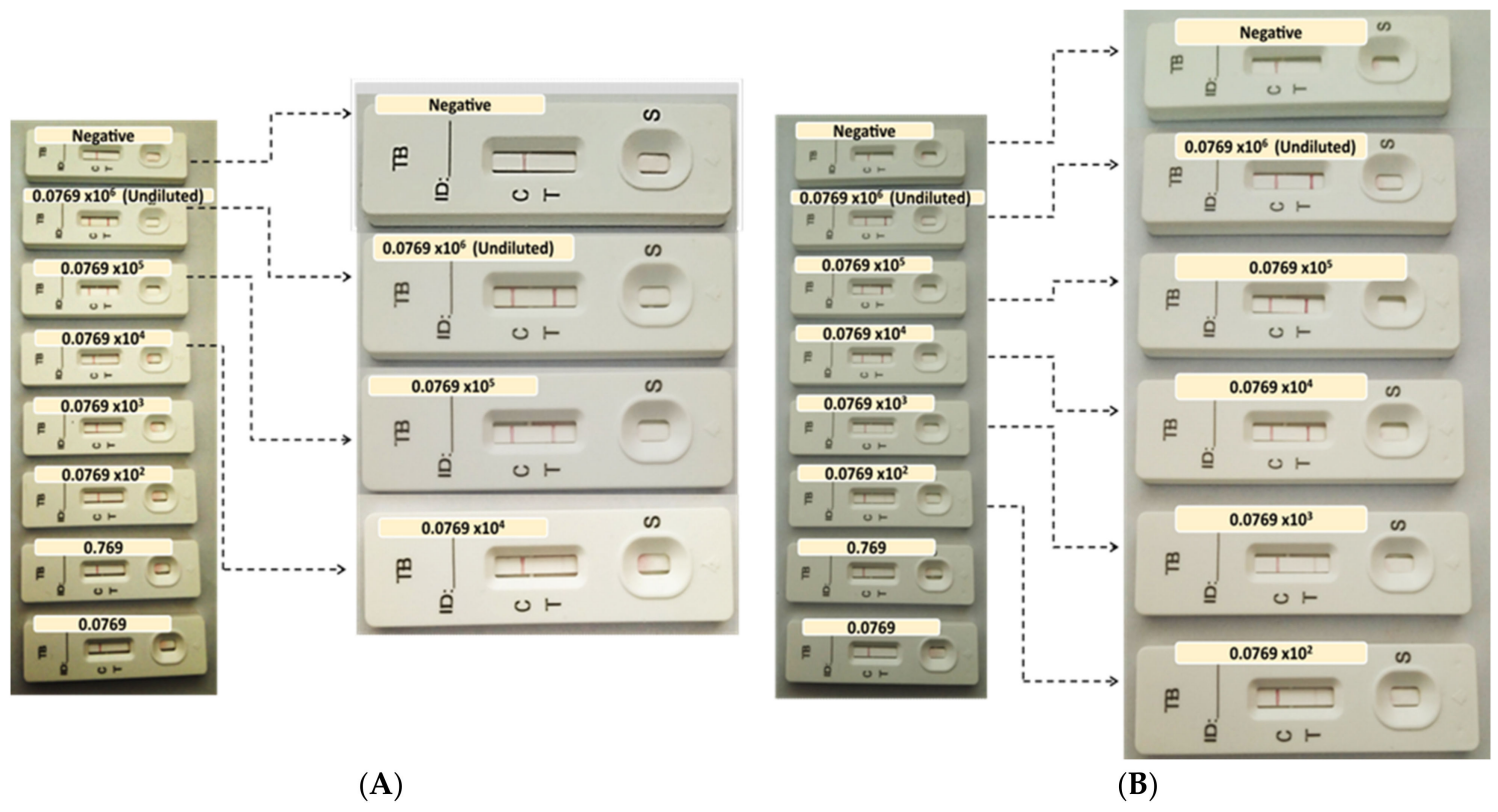
Determination of the LOD of CFP-10 (**A**) Ab1/Ab1 and (**B**) Ab1/Ab2 pairs. Ab1 was dispensed on the test line at a concentration of 0.5 mg/mL, and the anti-Mouse IgM antibody was dispensed at 0.3 mg/mL on the control line. The (**A**) Ab1 and (**B**) Ab2 were conjugated to AuNPs, then sprayed on the conjugate pad which was blocked using 3% BSA in sodium borate buffer. Following assembly of the nitrocellulose membrane, conjugate pad and absorbent pad on the backing cards, 3 mm strips were cut using the Kinbio cutter. Test samples were prepared at a range of 0.0769 × 10^6^ and 0.0769 ng/mL recombinant CFP-10 in running buffer. Samples were then applied at a volume of 65 µL and analyzed after 15 min of incubation. These results are representative of the same test performed in triplicate.

**Figure 5:**
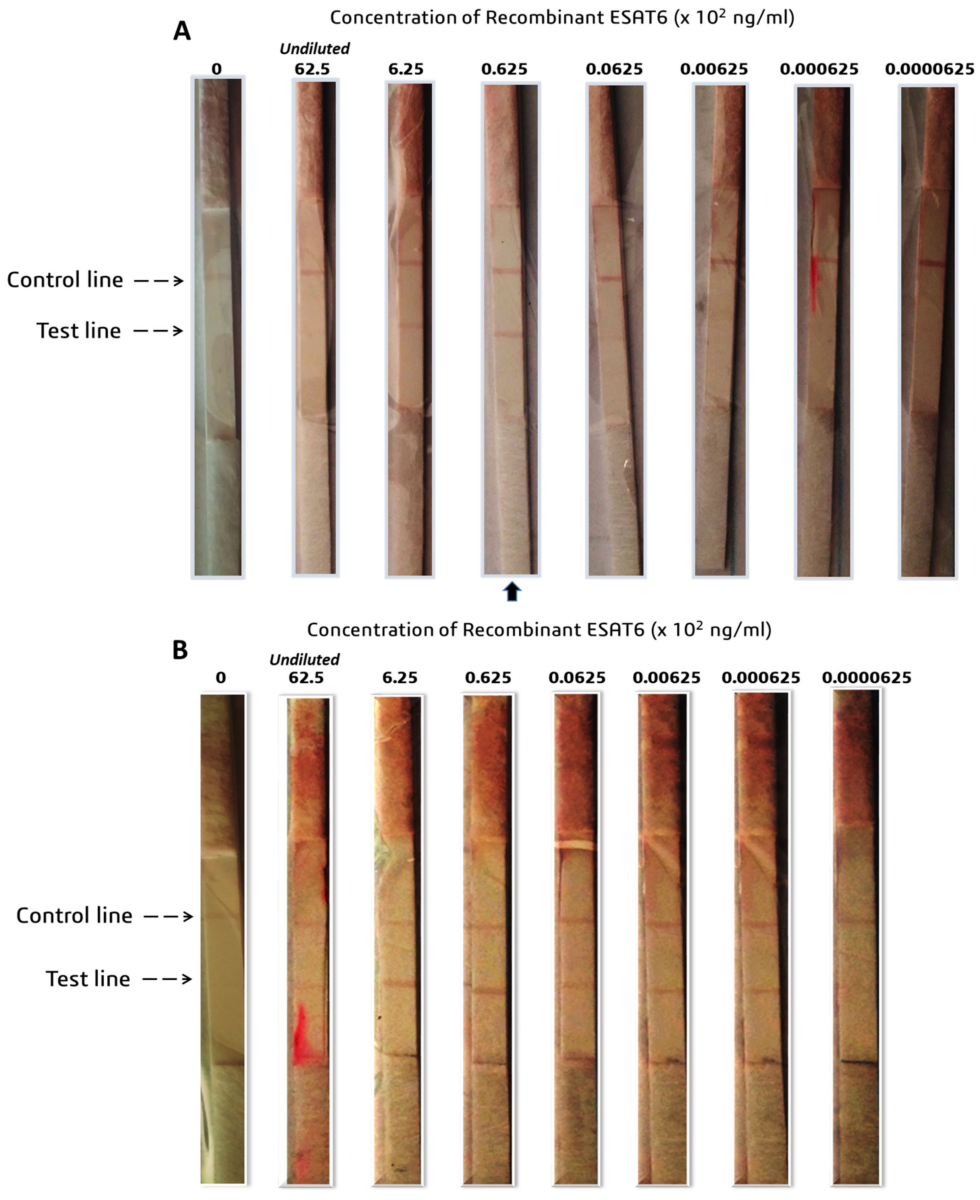
LOD of ESAT-6 (**A**) Ab6/Ab7 and (**B**) Ab7/Ab7 pair. (**A**) The Ab6 and (**B**) Ab7 capture antibodies were dispensed at 1 mg/mL on the nitrocellulose membrane, whilst the Ab7 conjugate was sprayed on the conjugate pad thereafter assembled on the backing cards. Recombinant ESAT-6 was serially diluted in the range indicated in order to compute the LOD. The arrows indicate the strip selected as the LOD. The results are representative of the same test performed in triplicate.

The visual LOD of the LFIA was determined on selected pairs, which showed the most consistent performance in the compatibility pairing studies: the Ab1/Ab1 and Ab1/Ab2 pairs for CFP-10 and Ab6/Ab7 and Ab7/Ab7 pairs for ESAT-6. The LOD for recombinant CFP-10 for the Ab1/Ab1 pair was 0.0769 × 10^4^ ng/mL (Figure 4A), and 0.0769 × 10^2^ ng/mL for the Ab1/Ab2 pair (Figure 4B). The Ab1/Ab2 pair was 100 times more sensitive than the Ab1/Ab1 pair, hence, it was chosen for future validations. ESAT-6 depicted an LOD of 62.5 ng/mL for the Ab6/Ab7 pair and 0.000625 ×10^2^ ng/mL for the Ab7/Ab7 pair (Figure 5A,B, respectively). The latter pair was 1000 times more sensitive to the ESAT-6 antigen and was thus selected for further studies. The CFP-10 and ESAT-6 prototypes showed potential for future clinical evaluations or studies, with LOD values of 7.69 and 0.0625 ng/mL, respectively. The ESAT-6 LFIA LOD was more than 120-fold lower compared to the CFP-10 LFIA.

## 4. Discussion

The attenuation of Mycobacterium bovis to the widely used BCG vaccine resulted in the deletion of the RD1 loci. We postulated that exploiting biomarkers within this region as targets for the TB-antigen RDT may minimize and/or eliminate false positives from previously BCG vaccinated individuals and/or those infected with other Mycobacteria species. The CFP-10 and ESAT-6 are two of the immunodominant antigens that are expressed by the RD1 loci and were explored as targets for the development of AuNP-based LFIA. The stability of antibodies with the 14 nm AuNPs at various pHs was tested, and overall the antibodies were stable at most pHs. Interestingly, Wu and colleagues found their CFP-10 and ESAT-6 antibodies to be suitable for conjugation with AuNPs sized between 18 and 20 nm [11], suggesting that the smaller-sized AuNPs are more stable with these biomarkers.

Initially, Dot spotting was used to determine compatible pairs of detection and capture antibodies for CFP-10 and ESAT-6 from different suppliers. Suitable antibody pairs will then be sourced from the same supplier to maintain consistency. In cases where the test line was visualized but with the faint control line, the antigen could be causing steric hindrance, blocking the binding site for the immobilized secondary antibody of the control line. The kinetics of solid-phase immobilized antibodies have demonstrated that equilibrium between antigen, capture and detection antibodies do not occur simultaneously, suggesting that steric hindrance plays a role in the detection-antigen-capture sandwich formation [22]. However in some instances, antibodies are able to re-organize to allow for further binding, and a high binding affinity may prevent this re-organization, causing saturation of binding sites at a faster rate [22,23,24]. Thus, in addition to target epitope compatibility, the association (k_ass_) and dissociation (k_off_) rate constants of the detection and capture antibodies may have a crucial role in defining suitable binding pairs.

The false negatives for the other pairs suggest that the capture and detection antibodies are competing for the same epitope on the antigen. It is possible that the detection antibody, which is the first to be encountered by the antigen in solution during testing, saturates the same epitope that is recognized and required for binding by the capture antibody. This effect was observed by Cavalera and colleagues, which they referred to as the ‘antigen hook effect’, and they showed that lowering the concentration of the detection antibody can enhance the sensitivity of LFIA. The ‘hook effect’ has been previously described and was initially observed for the LFIA-based pregnancy test, which measures the concentration of human chorionic gonadotropin (hCG). High concentrations of hCG led to disappearance of the test line, which yielded false negatives [25,26]. Cavalera et al. also reported that the distance of the test line from the sample pad affects the sensitivity of the LFIA. The further the test line was from the sample pad meant that there was an increase in contact time between the detection of the AuNP conjugate and the antigen which increased the saturation effect, thus augmenting the ‘antigen hook effect’ [27,28].

False positives were also observed when BSA-based buffers were used, such as when testing the Ab8/Ab8 pair in the 0.1 to 1% BSA concentration. Since BSA is a protein like the antigens, it may present with peptides that act as epitopes which may be causing non-specific binding. This effect became less pronounced at a BSA concentration of 0.1% versus at 1%. Using casein as an alternate buffer eliminated the false positives (results not shown), and this may be because casein is a less structured protein (mostly random coiled or disordered) than BSA (67% helix, 10% turns and 23% extended) [29,30], making it less stable and more prone to denaturation effects by the surfactant than BSA. It is possible that if a higher concentration of surfactant was used with the BSA buffers no false positives would appear. ‘Ghost lines’ are an artefact characterized by a red background encircling a white dot where an antibody was immobilized. The appearance of ‘ghost lines’ has been proposed to be as a result of high concentrations of capture antibody on the test line repelling the conjugate. Since only the Tris-based buffers caused the ‘ghost line’ effect with the Ab8/Ab8 pair, a plausible reason may be that the buffer confers an overall charge to the Ab8–antigen complex that caused it to be repelled by the immobilized capture antibody.

Although CFP-10’s Ab1/Ab1 pair showed potential, the Ab1/Ab2 pairing had an LOD that was 100 times more sensitive to the same antigen. Generally, for sandwich-type LFIA, a pair of mAbs targeting different epitopes of the antigen are often used over a single epitope-targeting mAb as both the capture and detection antibody [23,27,28]. The sensitivity of the LFIA is often lower with single epitope-targeting pairs. This does not mean that successful LFIAs have not been developed using the same mAb as both the capture and detection antibody, but some considerations and optimizations have to be performed [27,28]. Wu and colleagues determined the LOD of their CFP-10 test strip to be 2.4 ng/mL and 6.0 ng/mL for ESAT-6 [11], suggesting that the current study’s CFP-10 LFIA prototype is only slightly weaker in detecting the antigen. In contrast, the ESAT-6 prototype was 96-fold more sensitive than the one produced by Wu and colleagues, suggesting the prospect of a potentially better performance in clinical sample evaluation.

Other technologies have been developed to improve the sensitivity for detection of ESAT-6, such as the magnetic-bead-coupled AuNP-based immuno-PCR assay (MB GNP-I-PCR assay). Comparing the MB GNP-I-PCR assay to the ELISA, the authors reported an LOD of 10 fg/mL, which was 105-fold higher in sensitivity compared to the 1 ng/mL displayed by ELISA [31]. Simultaneously exploiting different sized AuNPs, 20 and 60 nm, to capture the ESAT-6 antigen may improve the LOD. However, this technique remains unsuitable for PoCs, as it requires additional steps that need skilled personnel and laboratory equipment and is more costly than the lateral flow assays. Previous studies have indicated that LOD values are not the ultimate predictor and/or determinant of sensitivity in clinical sample testing. Despite the reported LODs of 2.4 ng/mL and 6.0 ng/mL for CFP-10 and ESAT-6, respectively, Wu and colleagues found that the positive detection rate was 29.4% for CFP-10 and 41.2% for ESAT-6 in TB-positive plasma samples [11]. Accordingly, test conditions and LOD studies need to be carried out and optimized in the intended clinical sample of choice. Another type of immunoassay previously used for ESAT-6 detection in plasma/serum was electrochemiluminescence (ECL)-based, with a reported LOD of 6 pg/mL and 56% sensitivity [3]. There can be various contributing factors to this phenomenon, including the quantity of the biomarkers or analytes in the samples. For example, the status of expression and/or dysregulation of a potential biomarker is influenced by different factors, including the geography of the sampled population. Genetic variations in the host and of the Mycobacterium can contribute to the differential regulation and expression repertoire of biomarkers in the host [25,28]. Such factors need to be considered in biomarker discovery for TB.

Mehaffy and colleagues used mass spectroscopy (MS) to identify *M*. *tb* peptides in human serum. Their experimental approach included an exosome enrichment step from the plasma of TB-positive patients originating from four different countries: South Africa, Bangladesh, Peru and Vietnam. By using Multiple Reaction Monitoring MS, the group discovered that their representative peptides for CFP-10 and ESAT-6 were detected in 3% and 15% of the total sample population, respectively [32]. Out of the 10 South Africans, only one was smear positive who was also co-infected with HIV; none of these patients had the CFP-10 peptide in their serum, and the 3 who had the ESAT-6 peptide were also HIV positive. With respect to the South African cohort, these results were inconclusive because of the small sample size tested and due to that 90% of the samples that were collected were smear-negative samples [32].

In an independent study, Nanodisk-MS was used to detect CFP-10 and ESAT-6 in serum. The method involved the enrichment of selected CFP-10 and ESAT-6 peptides using antibody-conjugated Nanodisks prior to MS analysis. The LODs of the recombinant CFP-10 and ESAT-6 in the TB-free serum were 50 pM and 200 pM, respectively [14]. In HIV-negative TB patients’ serum, the sensitivity was 100% and 91% in smear-positive and smear-negative patients, respectively. The specificity was 87.1% in healthy controls and 90.6% in nontuberculous Mycobacteria patients. HIV co-infected TB patients exhibit EPTB, therefore, smear-negative and -positive EPTB and PTB patients were tested. In PTB cases the sensitivities were 91.3% and 82.4% in smear-positive and smear-negative patients, respectively, and 92.3% and 75.0% in EPTB patients [14]. The similarity in sensitivities between PTB and EPTB patients was an important outcome of the study, as it illustrated that serum has great potential to be used as an alternative sample for testing TB including EPTB. The key lesson learned from these studies is that a trend exists, wherein ESAT-6 shows a higher LOD compared to CFP-10 but with apparently superior sensitivity in clinical samples. Additionally, the specificity of the immunodominant antigens in healthy controls and more importantly in nontuberculous Mycobacteria patients is essential for developing a device that reduces cross-reactivity with nontuberculous Mycobacteria patients. These studies, including ours, demonstrated that nanotechnology-based immunoassays can greatly improve the simplicity, sensitivity and robustness of diagnostic methods.

## 5. Conclusions

The immunodominant antigens CFP-10 and ESAT-6 that are expressed by the RD1 loci of *M*. *tb* are potential biomarkers for developing a TB-antigen RDT that can discriminate previously active TB cases from previously BCG vaccinated and nontuberculous *Mycobacteria* cases. The pairs that were selected for preliminary testing with commercial recombinant protein in the current study showed great promise, with LODs that were either comparable or outperforming ones from those previously reported the in literature. An exhaustive study into biomarker discovery and research is required prior to choosing an appropriate target analyte in developing a diagnostic device for TB. The process is complicated and delayed by a spectrum of factors, such as the disease state, that is, active or latent disease, which can determine the level of expression and/or differential regulation of the targeted biomarker. Other factors such as the TB vaccination status, HIV status, age, and geography of the population tested need to be considered as well. Thus, future studies will explore other targets from the RD1 loci. Additionally, previous studies indicate that the methods used to improve sensitivity required additional steps, such as an enrichment step alongside exploiting immunoassays in combination with nanotechnology. These approaches need to be greatly refined in order to provide rapid results at the point of need/care, hence, our approach still has superiority. Proof-of-concept investigations for the rapid detection of TB in easily accessible clinical samples such as blood and saliva will be carried out both internally and subsequently validated externally to confirm clinical utility. This will greatly improve the diagnostic and therapeutic outcomes in low-income countries.

## Figures and Tables

**Table 1:** The primary antibodies used for CFP-10 and ESAT-6.

Antigen	Antibody Code	Antibody Details (Cat No.)	Supplier Details
CFP-10	Ab1	Mouse Monoclonal *Mycobacterium Tuberculosis* CFP-10 Antibody (KFB16)	Novus Biologicals, LLC (Briarwood Avenue, CO 80112, USA)
	Ab2	Mouse Monoclonal *Mycobacterium Tuberculosis* CFP-10 Antibody (KFB42)	Novus Biologicals, LLC
	Ab3	Mouse Anti-*Mycobacterium tuberculosis* CFP-10 Monoclonal Antibody (DMAB3941)	Creative diagnostics PTY (Ltd) (Shirley, NY, USA)
	Ab4	Mouse Anti-*Mycobacterium tuberculosis* CFP-10 Monoclonal Antibody (DMAB3943)	Creative diagnostics PTY (Ltd)
	Ab5	Rabbit Polyclonal Anti-CFP-10 antibody (AB45073)	Abcam PTY (LTD) (Discovery Drive, Cambridge, UK)
ESAT-6	Ab6	Mouse Anti-*Mycobacterium tuberculosis* ESAT-6 Monoclonal Antibody (DMAB3944)	Creative diagnostics PTY (Ltd)
	Ab7	Mouse Anti-*Mycobacterium tuberculosis* ESAT-6 Monoclonal Antibody (DMAB3945)	Creative diagnostics PTY (Ltd)
	Ab8	Rabbit Polyclonal Anti-ESAT-6 antibody (AB45074)	Abcam PTY (LTD)

**Table 2:** Summary of dot spotting analysis.

Capture Antibody	Control/Test	CFP-10					ESAT-6				
		Ab1	Ab2	Ab3	Ab4	Ab5	Ab6	Ab7	Ab8	Detector Antibody	
Ab1	Control	+++	++	++	+++						
	Test	+++	+++	-	+++						
Ab2	Control	++	-	+++	+++						
	Test	+++	-	+++	++						
Ab3	Control	+	+	++	++						
	Test	n/a	n/a	-	+++						
Ab4	Control	+	+	++	+						
	Test	-	-	++	-						
Ab5	Control					+++					
	Test					-					
Ab6	Control						++	+++			
	Test						-	-			
Ab7	Control						+++	+++			
	Test						-	++			
Ab8	Control								+++		
	Test								-		

Key: +++ high intensity; ++ moderate intensity; + low intensity/faint; - no dot visualized.

## Data Availability

Not applicable.
